# Actual over 10-year survival after liver resection for patients with intrahepatic cholangiocarcinoma

**DOI:** 10.18632/oncotarget.17815

**Published:** 2017-05-11

**Authors:** Anfeng Si, Jun Li, Hongjun Xiang, Shichao Zhang, Shilei Bai, Pinghua Yang, Xiaofeng Zhang, Yong Xia, Kui Wang, Zhenlin Yan, Wan Yee Lau, Lehua Shi, Feng Shen

**Affiliations:** ^1^ Department of Hepatic Surgery IV, The Eastern Hepatobiliary Surgery Hospital, Second Military Medical University, Shanghai, China; ^2^ Department of Hepatobiliary Surgery, The Lanzhou General Hospital of People's Liberation Army, Gansu, China; ^3^ Department of Minimally Invasive Surgery, The Eastern Hepatobiliary Surgery Hospital, Second Military Medical University, Shanghai, China; ^4^ Department of Clinical Database, The Eastern Hepatobiliary Surgery Hospital, Second Military Medical University, Shanghai, China; ^5^ Department of Hepatic Surgery II, The Eastern Hepatobiliary Surgery Hospital, Second Military Medical University, Shanghai, China; ^6^ Faculty of Medicine, The Chinese University of Hong Kong, Hong Kong, SAR, China

**Keywords:** intrahepatic cholangiocarcinoma, liver resection, long-term outcome, 10-year, actual survivors

## Abstract

Partial hepatectomy is a potentially curative therapy for intrahepatic cholangiocarcinoma (ICC). Unfortunately, the overall surgical prognosis remains dismal and the actual 10-year survival has not been reported. This study aimed to document 10-year actual survival rates, identify the prognostic factors associated with 10-year survival rate, and analyze the characteristics of patients who survived ≥ 10 years. Among 251 patients who underwent curative liver resection for ICC between 2003 and 2006 at the Eastern Hepatobiliary Surgery Hospital, 21 patients (8.4%) survived ≥ 10 years. The 5-, 7-, and 10-year overall survival rates were 32.3%, 22.3% and 8.4%, respectively. The 10-year cumulative incidence of ICC-related death and recurrence were 80.9% and 85.7%, respectively. Multivariate analysis based on competing risk survival analysis identified that tumor > 5 cm was independently associated with ICC-related death and recurrence (hazard ratios: 1.369 and 1.445, respectively), in addition to carcinoembryonic antigen (CEA) >10 U/mL, carbohydrate antigen 19-9 (CA19-9) >39 U/mL, multiple nodules, vascular invasion, nodal metastasis and local extrahepatic invasion. Patients who survived ≥ 10 years had a longer time to first recurrence, lower levels of CEA, CA19-9 and alkaline phosphatase, less perioperative blood loss, solitary tumor, smaller tumor size, and absence of nodal metastasis or local extrahepatic invasion. In conclusion, a 10-year survival after liver resection for ICC is possible and can be expected in approximately 8.4% of patients.

## INTRODUCTION

Intrahepatic cholangiocarcinoma (ICC) accounts for 10%-20% of primary hepatic malignancy, with its increasing global incidence only second to hepatocellular carcinoma (HCC) [1–4]. Most patients with unresectable ICC died within 6-12 months after disease diagnosis [5, 6], and liver resection is the only established therapy currently to achieve long-term survival in selected patients [7, 8]. Unfortunately, only about 20%-40% of patients with ICC are suitable for surgical resection and the overall surgical prognosis remains unsatisfactory [7]. The 5-year tumor recurrence rate has been reported to be 53%-79% and the corresponding survival rate 23.0%-35.2% [7, 9–17]. To date, the report on long-term survival after liver resection for ICC remains scarce, especially that based on follow-up of longer than 10 years. We only found one study that reported a 10-year survival of 23.1% in ICC patients after hepatic resection through the Kaplan–Meier survival analysis [18], which as we think might have overestimated the actual survival status for the biased censoring process associated with the Kaplan–Meier approach itself [19–21]. To the best of our knowledge, the actual 10-year follow-up and survival data in these patients have not been reported.

Of note, the risk factors associated with the prognosis in ICC patients after curative hepatectomy remain controversial [10–12, 15, 17, 18]. In particular, factors demonstrated as significant in some studies were found by others to be not significant. An example of this is tumor diameter, a few studies deemed it to be a non-significant factor associated with prognosis [11, 15, 17], but others have demonstrated its negative impact on survival in multivariate analysis [10, 12, 18]. It is thus difficult to accurately assess the true prognostic factors. Therefore, further studies with longer-term follow-up are required to better identify the survival factors and it is important to evaluate the characteristics of ICC patients who survived long-term after curative liver resection.

The aim of this study was to investigate the 10-year actual survival rates after initial hepatic resection in ICC patients, identify the characteristics of patients that have survived more than 10 years and discover the prognostic factors associated with long-term survival using competing risk survival analysis.

## RESULTS

### Baseline characteristics

Baseline clinicopathologic characteristics of all 251 patients are shown in Table 1. The median age of patients was 53 years (interquartile range [IQR], 46–62 years) and 160 (63.7%) patients are male. Among all patients, 23 (9.2%) patients had hepatolithiasis, 122 (48.6%) patients had positive hepatitis B surface antigen (HBsAg), 5 (2.0%) patients had positive anti-HCV, 49 (19.5%) patients had cirrhosis, and 171 (68.1%) patients had solitary tumor. The median tumor size was 5.4 cm (IQR, 4.0–8.0 cm). Seventy-seven (30.7%) patients underwent major liver resection, and nodal metastasis was found in 43 (17.1%) patients.

**Table 1:** Clinicopathologic characteristics (n = 251)

Variable	Number (%) / median (IQR)
**Age**, year	53.0 (46.0 – 62.0)
**Gender**	
Male	160 (63.7)
Female	91 (36.3)
**Hepatolithiasis**	
Yes	23 (9.2)
No	228 (90.8)
**HBsAg**	
Positive	122 (48.6)
Negative	129 (51.4)
**HBeAg**	
Positive	19 (7.6)
Negative	232 (92.4)
**Anti-HCV**	
Positive	5 (2.0)
Negative	246 (98.0)
**AFP**, μg/L	4.2 (2.4 – 15.3)
**CEA**, μg/L	2.1 (1.3 – 4.3)
**CA 19–9**, U/L	37.5 (13.7 – 196.9)
**TBIL**, μmol/L	13.5 (10.7 – 18.3)
**ALB**, g/L	42.6 (39.9 – 45.2)
**ALT**, U/L	31.3 (19.1 – 51.0)
**AST**, U/L	29.6 (23.2 – 43.7)
**ALP**, U/L	106.0 (80.0 – 147.0)
**PLT**, 10^9^/L	170.0 (133.0 – 219.0)
**PT**, seconds	11.9 (11.3 – 12.8)
**Child-Pugh grade**	
A	237 (94.4)
B	14 (5.6)
**Cirrhosis**	
Yes	49 (19.5)
No	202 (80.5)
**Tumor diameter**, cm	5.4 (4.0 – 8.0)
**Tumor number**	
Multiple	80 (31.9)
Solitary	171 (68.1)
**Vascular invasion**	
Presence	33 (13.1)
Absence	218 (86.9)
Nodal metastasis*	
Yes	43 (17.1)
No	208 (82.9)
**Local extrahepatic invasion**	
Yes	22 (8.8)
No	229 (91.2)
**Surgical margin**, cm	
> 1	64 (25.5)
≤ 1	187 (74.5)
**Tumor differentiation**	
Well	11 (4.4)
Moderate	208 (82.9)
Poor	32 (12.7)
**Perineural invasion**	
Yes	7 (2.8)
No	244 (97.2)
**Macroscopic type**	
MF	238 (94.8)
Non-MF	13 (5.2)
**Major hepatectomy**	
Yes	77 (30.7)
No	174 (69.2)
**Operative blood loss**, ml	300.0 (150.0 – 500.0)
**Blood transfusion**	
Yes	39 (15.5)
No	212 (84.5)
**Surgical complication**	
Yes	72 (28.7)
No	179 (71.3)
**Grade of complication**	
I/II	46 (63.9)
III/IV	26 (36.1)

Abbreviations: HBsAg, hepatitis B surface antigen; HBeAg, hepatitis B e antigen; HCV, hepatitis C virus; AFP, alpha-fetoprotein; CEA, carcinoembryonic antigen; CA19-9, carbonhydrateantigen19-9; TBIL, total bilirubin; ALB, albumin; ALT, alanine transaminase; AST, aspartate aminotransferase; ALP, alkaline phosphatase; PLT, platelet; PT, prothrombin time; MF, mass-forming; IQR, interquartile range.

*: nodes that were evaluated as normal and did not undergo dissection were defined as no metastasis in 98 patients.

### Tumor recurrence and overall survival

The median follow-up period was 32.1 months (IQR, 18.0–81.2 months). During the follow-up period, 220 (87.6%) patients developed tumor recurrence, and 165 of these patients developed recurrence within five years, 51 patients between five and ten years, and 4 patients beyond ten years after surgery. For the 238 patients (94.8%) who died during the study period, 170 patients died within five years, 60 patients between five and ten years, and 8 patients after ten years postoperatively. The remaining 13 patients (5.2%) were alive at the time of censor. Consequently, of all the 251 patients, 230 (91.6%) patients survived for less than, and 21 patients for more than 10 years after surgery.

Among the 13 survivors, 7 patients survived with tumor recurrence, and the remaining 6 patients were alive and free of recurrence. Among the 238 patients who died, 209 patients died of relapse, and another 4 patients who had recurrence but died of other reasons (hepatic failure, upper gastrointestinal bleeding, other malignant diseases and renal failure). The remaining 25 patients who did not have recurrence died of hepatic failures (n = 3), upper gastrointestinal bleeding (n = 1), other malignant diseases (n = 2), cardiovascular diseases (n = 4), respiratory diseases (n = 2), renal failure (n = 1), diabetic complication (n = 1), suicide (n = 1) and miscellaneous reasons (n = 10). Deaths that were caused by ICC recurrence or by other reasons were classified as ICC-related and non-ICC-related deaths, respectively. In this study, there were 209 ICC-related and 29 non-ICC-related deaths.

**Figure 1:**
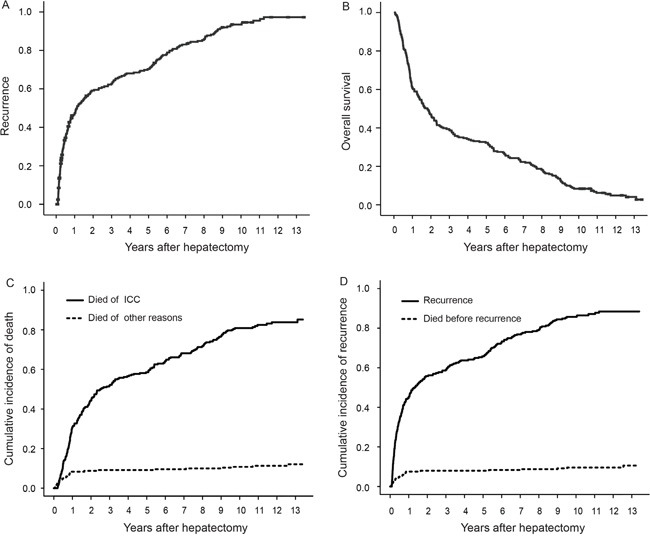
Recurrence, OS, cumulative incidence of ICC-related death and recurrence after curative hepatectomy **(A)** Recurrence; **(B)** OS; **(C)** Cumulative incidence of ICC-related death; **(D)** Cumulative incidence of recurrence.

The median time to recurrence (TTR) was 13.5 months (95% confidence interval [CI], 8.3–18.7 months) and the 5-, 7-, and 10-year tumor recurrence rates were 70.3%, 83.1% and 93.5%, respectively (Figure 1A). The median survival was 21.7 months (95% CI, 16.3–21.2 months) and the 5-, 7-, and 10-year overall survival (OS) rates were 32.3%, 22.3% and 8.4%, respectively (Figure 1B).

### Factors associated with ICC-related death

During the analysis, non-ICC-related deaths were considered as competing events for ICC-related deaths, and the 5-, 7- and 10-year cumulative incidences of ICC-related deaths were 58.6%, 68.2% and 80.9%, respectively (Figure 1C). The results of the univariate analysis on ICC-related deaths are shown in Supplementary Table 1. Multivariate analysis demonstrated that carcinoembryonic antigen (CEA) > 10 U/mL (hazard ratio [HR]: 1.812; 95% confidence interval [CI]: 1.180–2.784), carbohydrate antigen 19-9 (CA19-9) > 39 U/mL (1.711; 1.261–2.322), tumor diameter > 5 cm (1.369; 1.011–1.853), multiple tumors (1.614; 1.183–2.203), vascular invasion (1.847; 1.246–2.739), nodal metastasis (2.302; 1.552–3.413) and local extrahepatic invasion (2.284; 1.349–3.869) were significantly associated with ICC-related deaths (Table 2).

**Table 2:** Factors associated with cause-specific hazard of recurrence and ICC-related death according to multivariate Cox's Proportional Hazards Model

Variable	Tumor recurrence		Survival	
	HR (95% CI)	*p* value	HR (95% CI)	*p* value
**CEA**, μg/L, > 10 vs. ≤ 10	1.794 (1.166 – 2.761)	0.008	1.812 (1.180 – 2.784)	0.007
**CA 19–9**, U/L, > 39 vs. ≤ 39	1.641 (1.218 – 2.213)	0.001	1.711 (1.261 – 2.322)	0.001
**Tumor diameter**, cm, > 5 vs. ≤ 5	1.445 (1.080 – 1.933)	0.013	1.369 (1.011 – 1.853)	0.042
**Tumor number**, Multiple vs. Solitary	1.464 (1.089 – 1.968)	0.011	1.614 (1.183 – 2.203)	0.003
**Vascular invasion**, Yes vs. No	1.944 (1.317 – 2.868)	0.001	1.847 (1.246 – 2.739)	0.002
**Nodal metastasis**, Yes vs. No	2.034 (1.376 – 3.008)	<0.001	2.302 (1.552 – 3.413)	<0.001
**Local extrahepatic invasion**,Yes vs. No	1.933 (1.159 – 3.223)	0.012	2.284 (1.349 – 3.869)	0.002

Abbreviations: CEA, carcinoembryonic antigen; CA19-9, carbonhydrate antigen 19-9; HR, hazard ratio; CI, confidence interval.

### Factors associated with recurrence

Death before ICC recurrence was considered as a competing event for recurrence, and the 5-, 7- and 10-year cumulative incidences of ICC recurrence were 65.7%, 76.9% and 85.7%, respectively (Figure 1D). The results of the univariate analysis on ICC recurrence are shown in the Supplementary Table 1. Multivariate analysis identified that CEA > 10 U/mL (HR: 1.794; 95% CI: 1.166–2.761), CA19-9 > 39 U/mL (1.641; 1.218–2.213), tumor diameter > 5 cm (1.445; 1.080–1.933), multiple tumors (1.464; 1.089–1.968), vascular invasion (1.944; 1.317–2.868), nodal metastasis (2.034; 1.376–3.008) and local extrahepatic invasion (1.933; 1.159–3.223) were associated with recurrence (Table 2).

### Comparison of clinicopathologic data between patients who survived ≥ and < 10 years

Comparison of the clinicopathologic data between patients who survived ≥ and < 10 years is shownin Table 3. Whencompared with patients who survived < 10 years, patientswhosurvived ≥ 10 years had significantly lower levels of CEA (*p* = 0.018), CA19-9 (p = 0.020), and alkaline phosphatase (ALP, *p* = 0.002), smaller tumor size (p < 0.001), less operative blood loss (*p* = 0.001), and a lower incidence of multiple nodules (*p* = 0.040). No patient who survived ≥ 10 years had nodal metastasis and local extrahepatic invasion.

**Table 3:** Clinicopathologic characteristics between patients survived < or ≥ 10 years

Variable	Number (%) / median (IQR)		*p* value
	Survived < 10 years (n = 230)	Survived ≥ 10 years (n = 21)	
**Age**, year	53.0 (46.0 – 61.0)	56.0 (42.0 – 637.0)	0.608
**Gender**			
Male	146 (63.5)	14 (66.7)	0.771
Female	84 (36.5)	7 (33.3)	
**Hepatolithiasis**			
Yes	22 (9.6)	1 (4.8)	0.737
No	208 (90.4)	20 (95.2)	
**HBsAg**			
Positive	109 (47.4)	13 (61.9)	0.203
Negative	121 (52.6)	8 (38.1)	
**HBeAg**			
Positive	17 (7.4)	2 (9.5)	1.000
Negative	213 (92.6)	19 (90.5)	
**Anti-HCV**			
Positive	3 (1.3)	2 (9.5)	0.078
Negative	227 (98.7)	19 (90.5)	
**AFP**, μg/L	4.2 (2.4 – 15.5)	6.2 (2.3 – 12.7)	0.725
**CEA**, μg/L	2.3 (1.3 – 4.4)	1.7 (0.8 – 2.5)	0.018
**CA 19–9**, U/L	40.6 (13.9 – 246.1)	22.1 (9.0 – 35.5)	0.020
**TBIL**, μmol/L	13.5 (10.7 – 18.4)	12.1 (10.4 – 15.7)	0.273
**ALB**, g/L	42.6 (39.8 – 45.1)	44.6 (41.0 – 46.5)	0.055
**ALT**, U/L	31.2 (19.2 – 49.9)	31.8 (18.6 – 57.3)	0.800
**AST**, U/L	29.6 (23.4 – 46.2)	31.9 (20.3 – 41.0)	0.569
**ALP**, U/L	108.0 (82.0 – 151.3)	84.0 (68.0 – 101.5)	0.002
**PLT**, 10^9^/L	168.5 (133.0 – 219.0)	171.0 (141.0 – 196.5)	0.995
**PT**, seconds	11.9 (11.3 – 12.8)	12.1 (11.6 – 13.1)	0.333
**Child-Pugh grade**			
A	216 (93.9)	21 (100.0)	0.284
B	14 (6.1)	0 (0.0)	
**Cirrhosis**			
Yes	45 (19.6)	4 (19.0)	1.000
No	185 (80.4)	17 (81.0)	
**Tumor diameter**, cm	5.5(4.2 – 8.0)	3.8 (2.4 – 5.0)	< 0.001
**Tumor number**			
Multiple	78 (33.9)	2 (9.5)	0.040
Solitary	152 (66.1)	19 (90.5)	
**Vascular invasion**			
Presence	32 (13.9)	1 (4.8)	0.395
Absence	198 (86.1)	20 (95.2)	
**Nodal metastasis**			
Yes	43 (18.7)	0 (0.0)	0.030
No	187 (81.3)	21 (100.0)	
**Local extrahepatic invasion**			
Yes	22 (9.6)	0 (0.0)	0.231
No	208 (90.4)	22 (100.0)	
**Surgical margin**, cm			
> 1	57 (24.8)	7 (33.3)	0.389
≤ 1	173 (75.2)	14 (66.7)	
**Tumor differentiation**			
Well	9 (3.9)	2 (9.5)	0.540
Moderate	192 (83.5)	16 (76.2)	
Poor	29 (12.6)	3 (14.3)	
**Perineural invasion**			
Yes	7 (3.0)	0 (0.0)	1.000
No	223 (97.0)	21 (100.0)	
**Macroscopic type**			
MF	217 (94.3)	21 (100.0)	0.609
Non-MF	13 (5.7)	0 (0.0)	
**Major hepatectomy**			
Yes	72 (31.3)	5 (23.8)	0.476
No	158 (68.7)	16 (76.2)	
**Operative blood loss**, ml	300.0 (200.0 – 500.0)	150.0 (100.0 – 350.0)	0.011
**Blood transfusion**			
Yes	38 (16.5)	1 (4.8)	0.267
No	192 (83.5)	20 (95.2)	
**Surgical complication**			
Yes	67 (29.1)	5 (23.8)	0.606
No	163 (70.9)	16 (76.2)	
**Grade of complication**			
I/II	43 (64.2)	3 (60.0)	1.000
III/IV	24 (35.8)	2 (40.0)	

Abbreviations: HBsAg, hepatitis B surface antigen; HBeAg, hepatitis B e antigen; HCV, hepatitis C virus; AFP, alpha-fetoprotein; CEA, carcinoembryonic antigen; CA19-9, carbonhydrateantigen19-9; TBIL, total bilirubin; ALB, albumin; ALT, alanine transaminase; AST, aspartate aminotransferase; ALP, alkaline phosphatase; PLT, platelet; PT, prothrombin time; MF, mass-forming; HR, hazard ratio; CI, confidence interval.

In addition, comparison of the clinicopathologic characteristics of the patients who survived 5-10 years and those who survived ≥ 10 years revealed that the latter group of patients had significantly smaller tumor size (p = 0.049). There were no significant differences in the other baseline characteristics (Supplementary Table 2).

### Treatment for recurrence between patients who survived ≥ and < 10 years

For patients who survived < 10 years, 206 patients had recurrences, in which 114 patients developed recurrences within 1 year, 49 patients between 2 and 5 years, and only 43 patients developed recurrences between 5 and 10 years postoperatively. Among all these patients who developed recurrences, 127 (61.6%) patients had intrahepatic recurrence only, 36 (17.5%) patients had extrahepatic metastases only, and 43 (20.9%) patients had intra- and extrahepatic recurrent diseases. The treatment for patients with recurrences consisted of re-hepatectomy (n = 14), percutaneous radiofrequency ablation (PRFA, n = 28), transarterial chemoembolization (TACE, n = 35), chemotherapy (n = 47), and conservative therapy (n = 82).

Among the patients who survived ≥ 10 years, 14 patients had recurrences during follow-up, in which 2 patients developed recurrences within 5 years, 8 patients between 5 and 10 years, and the remaining 4 patients beyond 10 years postoperatively. Among these 14 patients, 12 (85.8%) patients had intrahepatic recurrence only, 1 (7.1%) patient had extrahepatic metastases only, and 1 (7.1%) patient had intra- and extrahepatic recurrent diseases. The treatment for recurrences consisted of re-hepatectomy (n = 6), PRFA (n = 4), TACE (n = 2), chemotherapy (n = 1), and conservative therapy (n = 1). Among the 21 patients who survived ≥ 10 years, 8 patients died of ICC recurrence (n = 7) or cardiovascular diseases (n = 1) beyond ten years after surgery. Seven and 6 of the remaining 13 patients survived with and without tumor recurrence respectively, on the date when this study was censored.

### Clinicopathologic features of the 10-year recurrence-free survivors

Table 4 shows the clinicopathologic features of the 10-year recurrence-free survivors (cases 1–7). Of these 7 patients, 6 (case 2–7) patients were still alive on the date when the study was censored and 1 (case 1) patient died of cardiovascular disease. Six (case 1–6) patients were males. All the 7 survivors had Child-Pugh grade A liver function and they had no cirrhosis. Four of these 7 patients were HBsAg positive. Histopathological examination showed that all the 7 patients had a solitary moderately differentiated tumor with a diameter which ranged from 2.0 to 5.0 cm. There were no evidences of nodal metastasis or local extrahepatic invasion in these 7 patients.

**Table 4:** Clinicopathological data of 10-year recurrence-free survivors

Case	Age(years)	Sex	HBsAg	Cirrhosis	Child-Puge	Tumor diameter (cm)	Tumor number	Differentiation	Nodal metastasis	VI*	OS(monthes)	Outcome
1	58	M	Positive	No	A	2.0	Solitary	Moderate	No	No	150.6	Dead
2	68	M	Positive	No	A	2.2	Solitary	Moderate	No	Yes	122.9	Alive
3	60	M	Positive	No	A	2.5	Solitary	Moderate	No	No	160.8	Alive
4	41	M	Negative	No	A	3.0	Solitary	Moderate	No	No	121.5	Alive
5	50	M	Negative	No	A	4.0	Solitary	Moderate	No	No	139.6	Alive
6	59	M	Negative	No	A	5.0	Solitary	Moderate	No	No	123.3	Alive
7	56	F	Positive	No	A	2.5	Solitary	Moderate	No	No	127.1	Alive

Abbreviation: ICC, intrahepatic cholangiocarcinoma; VI, vascular invasion; OS, overall survival; M, male; F, female.

*: vascular invasion in this patient is microscopic vascular invasion.

## DISCUSSION

Liver resection has been the only curative therapy in selected patients with ICC [7, 8]. So far, no published study with enough follow-up time is available to provide robust actual 10-year outcomes in ICC patients following curative resection. To the best of our knowledge, the present study has provided an actual 10-year OS of 8.4% in ICC patients after curative resection through actual follow-up.

A recent study by Spolverato G *et al* reported a cure model to define a statistical cure for ICC and estimated the probability of being cured after liver resection, which indicated that a statistical cure was possible in some surgically treated patients with ICC [22]. However, the model can be considered useful mathematically only when it is believed that a cure fraction exists [23, 24]. In our study, a proportion of ICC patients did experience a plateauing of survival after a certain period of time, which echoed the results from Spolverato G *et al* and also indicated that cure was indeed possible in selected ICC patients after hepatectomy.

The Kaplan-Meier analysis frequently used to measure outcome risks over time provides a nonparametric estimate of the overall survival probability of an event of interest. However, the process of censoring associated with this method might lead to a bias and a gross overestimation of survival [19–21]. In addition, ignoring competing events in the statistical analysis can easily lead to flawed results and conclusions [22]. Therefore, cumulative incidences of recurrence and ICC-related death were used in the present study to measure outcome risks after a long-term follow up [25, 26]. We found that the 10-year cumulative incidence of recurrence was notably 80.2% and the cumulative ICC-related mortality was 80.9% at 10 years. In this study, although most tumor recurrences developed within 5 years after surgery, some patients still developed recurrences thereafter. Surprisingly, recurrence of ICC may occur even after a 10-year recurrence-free period: four patients who survived ≥ 10 years in our study developed recurrence beyond 10 years after curative surgery. Whether the delayed recurrence in long-term survivors was likely to be the result from *de novo* ICC or the progression of occult metastatic diseases still warrants validation by future studies [27, 28]. Nevertheless, this study has indicated that tumor recurrence was a persistent problem and the leading cause of death for ICC patients after surgery. Although how long a follow-up period after surgery should be remains to be determined, our data supported the importance of a very long-term follow-up, even lifetime, for ICC patients after liver resection.

In the present study, the predictive analysis of variables associated with prognosis was conducted using the competing risks model. The results showed that CEA > 10 U/mL, CA19-9 > 39 U/mL, tumor diameter > 5 cm, multiple tumors, vascular invasion, nodal metastasis and local extrahepatic invasion were closely associated with recurrence and ICC-related death. The result was consistent with the outcomes reported from other studies [9–14]. In addition, our data also demonstrated that tumor diameter > 5 cm was an independent risk factor associated with prognosis, despite the conflicting results from the previous studies with regard to the association between tumor diameter and prognosis after curative surgery for ICC [10–12, 15, 17, 18]. One explanation could be that previous studies did not specifically consider the confounding of competing events and did not have long-term postoperative follow-up.

This study specifically investigated the patients with ICC who have survived more than 10 years after surgery, and found that these patients experienced recurrence later and were more likely to have smaller and solitary tumor without any nodal metastasis or local extrahepatic invasion, as well as lower CEA, CA19-9 and ALP levels and less operative blood loss. Although the distribution of the clinicopathologic factors in this study reflected the heterogeneity of the population, it indicated that the presence of the factors mentioned above could preclude long-term survival after curative surgery and these patients clearly deserve rigorous and regular follow-up.

Our study had some limitations. First, given its retrospective nature, this study is inevitably exposed to selection bias. Second, the 10-year survivors were relatively small in number for a more robust statistical analysis. Therefore, further studies on a larger number of patients are necessary to evaluate the prognostic factors of ICC more accurately.

In conclusion, 10-year survival after liver resection for ICC is possible and can be expected in approximately 8.4% of patients. Recurrence of ICC might occur even after a 10-year recurrence-free period. Tumor diameter was a risk factor of long-term prognoses. In addition, long-term or even life-long follow-up after resection of ICC is crucial.

## MATERIALS AND METHODS

### Patients

A total of 313 consecutive patients who underwent partial hepatectomy for pathologically proven ICC between April 2003 and August 2006 at the Department of Hepatic Surgery of the Eastern Hepatobiliary Surgery Hospital (EHBH) were enrolled into the study. Patients met the following criteria: (1) no previous history of other malignancies; (2) had not received any anti-cancer therapy before surgery; (3) underwent a R0 resection, defined as complete removal of macroscopic nodules and absence of microscopic disease at the surgical margin [9]; (4) had not died within 30 days from the date of surgery, and (5) had complete clinical data and follow-up information. Finally, 251 patients were included for further analysis.

This study was approved by the Institutional Ethics Committee of the EHBH. Informed consent was obtained from all the patients before surgery for their data to be used for research.

### Preoperative examination and surgical procedure

After a detailed history and a complete physical examination, all patients were tested for liver and renal functions, hepatitis B and C serology, CA19-9, CEA, α-fetoprotein, abdominal ultrasound, chest x-ray, contrast-enhanced magnetic resonance imaging (MRI) and/or computed tomography (CT) scan of the abdomen. A preoperative diagnosis of ICC was mainly based on the combination of the serological and radiological findings [15], and on the clinical manifestations which were different from HCC and other metastatic malignancies. The lymph node status was evaluated with contrast-enhanced CT scans or MRI.

Partial hepatectomies were performed on patients with good general condition and liver functional reserve, technically resectable tumor and sufficient estimated volume of future liver remnant, and without any evidences of distant tumor metastases [10]. Perihepatic lymph node adenopathy on preoperative CT scan or MRI was considered primarily for operation as long as the nodes could be completely and safely resected, which was judged by experienced surgeons. Liver resection of three or more segments was defined as major resection. Any additional nodules, which were found intraoperatively, were also resected if the surgeon considered the resection to be safe. Local extrahepatic invasions, defined as direct invasion of any adjacent organs and structures, which included the colon, duodenum, stomach, common bile duct, retrohepatic vena cava, abdominal wall and diaphragm, were also resected if the surgeon considered the resection to be safe. Dissection of perihepatic lymph nodes was only carried out in patients who were preoperatively or intraoperatively diagnosed as ICC based on imaging, serology and frozen section results. Histopathological study of the resected specimens was performed independently by three pathologists and disagreements were resolved through discussion until a consensus was reached [29]. Perioperative complications were observed and defined as grade I-IV based on the previously reported classification system [30].

### Follow-up and endpoints

Patients were followed-up once every 2 months in the first 2 years after surgery and once every 3 months thereafter. At each follow-up visit, serum CEA and CA19-9 levels, liver function test, and abdominal ultrasound were performed routinely. Contrast-enhanced CT or MRI was performed once every 3-6 months or earlier if tumor recurrence or metastasis was suspected. Bone metastases were detected by scintigraphy or positron emission tomography (PET). ICC recurrence was diagnosed based on the combined findings of the above examinations. Deaths were classified as “ICC-related death” and “non-ICC-related death” which were caused by other reasons, including hepatic failures, upper gastrointestinal bleeding, other malignant diseases, cardiovascular diseases, renal failure, diabetic complication, or other causes.

OS and TTR were used as end points. OS was defined as the interval between the date of surgery and the date of patient death or last follow-up. TTR was the interval between the date of surgery and the date when recurrence/metastasis was diagnosed. This study was censored on October 25, 2016.

### Statistical analysis

Continuous variables were initially explored for normal distribution using the Shapiro-Wilk test. As a normal distribution could not be confirmed for most variables, the variables were expressed as median and IQR. Differences between the groups were compared by the *t*-test or Mann-Whitney *U* test. Categorical variables were compared using the χ^2^ test, Yates’ correction test or Fisher's exact test. The collinearity was assessed by examining the variance inflation factors, and no collinearity was found between the variables. Survival curves were estimated by the Kaplan–Meier method.

Cumulative incidences of recurrence and ICC-related death were estimated while taking into account the competing risk of non-ICC-related death using a competing risk analysis as defined by Fine and Gray [31]. Predictive analysis of variables associated with the cause-specific hazard of recurrence and ICC-related death was done using the univariate and multivariate Cox proportional hazard model [31].

All statistical analyses were performed using SPSS 19.0 for Windows (SPSS, Chicago, IL) and R software v. 2.15.3 (R Foundation for Statistical Computing, Vienna, Austria; www.r-project.org). All reported *p* values were two-sided, and *p* < 0.05 was considered statistically significant.

## SUPPLEMENTARY MATERIALS TABLES





## Abbreviations

ICC: intrahepatic cholangiocarcinoma
CEA: carcinoembryonic antigen
CA19-9: carbohydrate antigen 19-9
HCC: hepatocellular carcinoma
IQR: interquartile range
HBsAg: hepatitis B surface antigen
HCV: hepatitis C virus
TTR: time to recurrence
CI: confidence interval
OS: overall survival
HR: hazard ratio
ALP: alkaline phosphatase
PRFA: percutaneous radiofrequency ablation
TACE: transarterial chemoembolization
MRI: magnetic resonance imaging
CT: computed tomography
PET: positron emission tomography
