# Supplementary material for: Actual over 10-year survival after liver resection for patients with intrahepatic cholangiocarcinoma
Source: Oncotarget. 2017 May 11;8(27):44521–32. doi: 10.18632/oncotarget.17815 (PMC5546499; doi:10.18632/oncotarget.17815)
Supplement: Supplementary file 1 [file oncotarget-08-44521-s001.pdf]

Actual over 10-year survival after liver resection for patients with intrahepatic cholangiocarcinoma

SUPPLEMENTARY MATERIALS

SUPPLEMENTARY TABLES

Supplementary Table 1: Factors associated with cause-specific hazard of recurrence and ICC-related death according to the univariate Cox's Proportional Hazards Model

See Supplementary File 1

Supplementary Table 2: Clinicopathologic characteristics between patients survived 5-10 or ≥ 10 years

See Supplementary File 1
