# Supplementary material for: Actual over 10-year survival after liver resection for patients with intrahepatic cholangiocarcinoma
Source: Oncotarget. 2017 May 11;8(27):44521–32. doi: 10.18632/oncotarget.17815 (PMC5546499; doi:10.18632/oncotarget.17815)
Supplement: Supplementary file 2 [file oncotarget-08-44521-s002.doc]

**Supplementary Table 1. Factors associated with cause-specific hazard of recurrence and ICC-related death according to the univariate Cox’s Proportional Hazards Model**

| **Variable** | **Tumor recurrence** | |  | **Survival** | |
| --- | --- | --- | --- | --- | --- |
|  | **HR (95%CI)** | ***p* value** |  | **HR (95%CI)** | ***p* value** |
| **Age,** year, > 55 vs. ≤ 55 | 0.805 (0.614 – 1.056) | 0.117 |  | 0.851 (0.645 – 1.124) | 0.256 |
| **Gender,** Male vs. Female | 0.894 (0.679 – 1.175) | 0.421 |  | 0.917 (0.692 – 1.214) | 0.545 |
| **Hepatolithiasis,** Yes vs. No | 1.319 (0.790 – 2.202) | 0.290 |  | 1.323 (0.781 – 2.241) | 0.298 |
| **HBsAg,** Positive vs. Negative | 0.812 (0.623 – 1.059) | 0.125 |  | 0.739 (0.562 – 0.973) | 0.031 |
| **HBeAg,** Positive vs. Negative | 1.040 (0.615 – 1.758) | 0.884 |  | 1.028 (0.596 – 1.772) | 0.920 |
| **Anti-HCV,** Positive vs. Negative | 0.371 (0.118 – 1.165) | 0.089 |  | 0.457 (0.146 – 1.432) | 0.179 |
| **AFP,** µg/L, > 20 vs. ≤ 20 | 1.180 (0.858 – 1.623) | 0.310 |  | 1.176 (0.849 – 1.629) | 0.328 |
| **CEA,** µg/L, > 10 vs. ≤ 10 | 2. 453 (1.606 – 3.747) | <0.001 |  | 2.554 (1.676 – 3.892) | <0.001 |
| **CA 19-9,** U/L, > 39 vs. ≤ 39 | 2.036 (1.542 – 2.689) | <0.001 |  | 2.164 (1.627 – 2.877) | <0.001 |
| **TBIL,** μmol/L, > 17.1 vs. ≤ 17.1 | 1.163 (0.872 – 1.552) | 0.304 |  | 1.262 (0.940 – 1.693) | 0.122 |
| **ALB,** g/L, > 35 vs. ≤ 35 | 0.473 (0.267 – 0.837) | 0.010 |  | 0.380 (0.214 – 0.674) | 0.001 |
| **ALT,** U/L, > 44 vs. ≤ 44 | 1.058 (0.791 – 1.415) | 0.704 |  | 0.987 (0.732 – 1.330) | 0.932 |
| **AST,** U/L, > 45 vs. ≤ 45 | 1.225 (0.892 – 1.683) | 0.210 |  | 1.344 (0.974 – 1.885) | 0.072 |
| **ALP,** U/L, > 129 vs. ≤ 129 | 1.554 (1.176 – 2.054) | 0.002 |  | 1.763 (1.328 – 2.340) | <0.001 |
| **PLT,** 10^9^/L, < 100 vs. ≥ 100 | 0.851 (0.560 – 1.293) | 0.450 |  | 0.859 (0.561 – 1.316) | 0.485 |
| **PT,** seconds, > 13 vs. ≤ 13 | 1.128 (0.812 – 1.566) | 0.473 |  | 1.181 (0.846 – 1.647) | 0.329 |
| **Child-Pugh classification,** A vs. B | 0.509 (0.282 – 0.918) | 0.025 |  | 0.452 (0.250 – 0.817) | 0.009 |
| **Cirrhosis,** Yes vs. No | 0.981 (0.706 – 1.364) | 0.910 |  | 0.902 (0.644 – 1.262) | 0.546 |
| **Tumor diameter,** cm, > 5 vs. ≤ 5 | 1.957 (1.396 – 2.412) | <0.001 |  | 1.994 (1.511 – 2.632) | <0.001 |
| **Tumor number,** Multiple vs. Solitary | 1.787 (1.340 – 2.385) | <0.001 |  | 1.959 (1.457 – 2.634) | <0.001 |
| **Vascular invasion,**  Presence vs. Absence | 1.799 (1.228 – 2.637) | 0.003 |  | 1.926 (1.309 – 2.833) | 0.001 |
| **Nodal metastasis,** Yes vs. No | 2.863 (1.952 – 4.198) | <0.001 |  | 3.280 (2.223 – 4.840) | <0.001 |
| **Local extrahepatic invasion,**  Yes vs. No | 2.516 (1.532 – 4.133) | <0.001 |  | 2.513 (1.511 – 4.178) | <0.001 |
| **Surgical margin**, cm, > 1 vs. ≤ 1 | 0.824 (0.608 – 1.117) | 0.213 |  | 0.787 (0.576 – 1.075) | 0.133 |
| **Poor tumor differentiation,**  Yes vs. No | 1.213 (0.820 – 1.795) | 0.333 |  | 1.226 (0.823 – 1.828) | 0.316 |
| **Perineural invasion,** Yes vs. No | 1.178 (0.484 – 2.869) | 0.718 |  | 1.600 (0.657 – 3.897) | 0.300 |
| **Macroscopic type,** non-MF vs. MF | 2.276 (1.234 – 4.198) | 0.008 |  | 2.171 (1.178 – 4.003) | 0.013 |
| **Major hepatectomy**, yes vs. no | 1.107 (0.829 – 1.478) | 0.492 |  | 1.137 (0.845 – 1.531) | 0.397 |
| **Operative blood loss,** ml  > 500 vs. ≤ 500 | 1.477 (1.084 – 2.011) | 0.013 |  | 1.529 (1.114 – 2.097) | 0.009 |
| **Blood transfusion,** Yes vs. No | 1.570 (1.099 – 2.243) | 0.013 |  | 1.753 (1.224 – 2.512) | 0.002 |
| **Surgical complication,** Yes vs. No | 1.002 (0.745 – 1.348) | 0.987 |  | 1.123 (0.832 – 1.516) | 0.450 |

**Abbreviations:** HBsAg, hepatitis B surface antigen; HBeAg, hepatitis B e antigen; HCV, hepatitis C virus; AFP, alpha-fetoprotein; CEA, carcinoembryonic antigen; CA19-9, carbonhydrateantigen19-9; TBIL, total bilirubin; ALB, albumin; ALT, alanine transaminase; AST, aspartate aminotransferase; ALP, alkaline phosphatase; PLT, platelet; PT, prothrombin time; MF, mass-forming; HR, hazard ratio; CI, confidence interval.

**Supplementary Table 2. Clinicopathologic characteristics between patients survived 5-10 or** ≥ 10 years

| **Variable** | **Number (%) / median (IQR)** | | ***p* value** |
| --- | --- | --- | --- |
|  | **Survived 5-10 years (n = 60)** | **Survived** ≥ **10 years (n = 21)** |  |
| **Age,** year | 55.0 (48.0 – 63.0) | 56.0 (42.0 – 63.0) | 0.859 |
| **Gender** |  |  |  |
| Male | 38 (63.3) | 14 (66.7) | 0.771 |
| Female | 22 (36.7) | 7 (33.3) |  |
| **Hepatolithiasis** |  |  |  |
| Yes | 2 (3.3) | 1 (4.8) | 1.000 |
| No | 58 (96.7) | 20 (95.2) |  |
| **HBsAg** |  |  |  |
| Positive | 38 (63.3) | 13 (61.9) | 0.907 |
| Negative | 22 (36.7) | 8 (38.1) |  |
| **HBeAg** |  |  |  |
| Positive | 3 (5.0) | 2 (9.5) | 0.830 |
| Negative | 57 (95.0) | 19 (90.5) |  |
| **Anti-HCV** |  |  |  |
| Positive | 1 (1.7) | 2 (9.5) | 0.332 |
| Negative | 59 (98.3) | 19 (90.5) |  |
| **AFP,** µg/L | 4.3 (2.9 – 14.6) | 6.2 (2.3 – 12.7) | 0.953 |
| **CEA,** μg/L | 1.9 (1.2 – 3.1) | 1.7 (0.8 – 2.5) | 0.278 |
| **CA 19-9,** U/L | 24.5 (11.5 – 45.6) | 22.1 (9.0 – 35.5) | 0.575 |
| **TBIL,** μmol/L | 14.6 (10.7 – 18.2) | 12.1 (10.4 – 15.7) | 0.311 |
| **ALB,** g/L | 43.4 (41.4 – 46.3) | 44.6 (41.0 – 46.5) | 0.586 |
| **ALT,** U/L | 27.8 (18.7 – 46.6) | 31.8 (18.6 – 57.3) | 0.800 |
| **AST,** U/L | 29.0 (22.4 – 39.4) | 31.9 (20.3 – 41.0) | 0.441 |
| **ALP,** U/L | 99.5 (72.5 – 134.0) | 84.0 (68.0 – 101.5) | 0.087 |
| **PLT,** 10^9^/L | 162.5 (130.8 – 200.0) | 171.0 (141.0 – 196.5) | 0.383 |
| **PT,** seconds | 11.8 (11.3 – 12.5) | 12.1 (11.6 – 13.1) | 0.135 |
| **Child-Pugh grade** |  |  |  |
| A | 59 (98.3) | 21 (100.0) | 1.000 |
| B | 1 (1.7) | 0 (0.0) |  |
| **Cirrhosis** |  |  |  |
| Yes | 15 (25.0) | 4 (19.0) | 0.799 |
| No | 45 (75.0) | 17 (81.0) |  |
| **Tumor diameter,** cm | 4.9 (3.4 – 6.7) | 3.8 (2.4 – 5.0) | 0.049 |
| **Tumor number** |  |  |  |
| Multiple | 10 (16.7) | 2 (9.5) | 0.663 |
| Solitary | 50 (83.3) | 19 (90.5) |  |
| **Vascular invasion** |  |  |  |
| Presence | 4 (6.7) | 1 (4.8) | 1.000 |
| Absence | 56 (93.3) | 20 (95.2) |  |
| **Nodal metastasis** |  |  |  |
| Yes | 1 (1.7) | 0 (0.0) | 1.000 |
| No | 59 (98.3) | 21 (100.0) |  |
| **Local extrahepatic invasion** |  |  |  |
| Yes | 1 (1.7) | 0 (0.0) | 1.000 |
| No | 59 (98.3) | 22 (100.0) |  |
| **Surgical margin,** cm |  |  |  |
| > 1 | 20 (33.3) | 7 (33.3) | 0.389 |
| ≤ 1 | 40 (66.7) | 14 (66.7) |  |
| **Tumor differentiation** |  |  |  |
| Well | 7 (11.7) | 2 (9.5) | 0.852 |
| Moderate | 47 (78.3) | 16 (76.2) |  |
| Poor | 6 (10.0) | 3 (14.3) |  |
| **Perineural invasion** |  |  |  |
| Yes | 1 (1.7) | 0 (0.0) | 1.000 |
| No | 59 (98.3) | 21 (100.0) |  |
| **Macroscopic type** |  |  |  |
| MF | 59 (98.3) | 21 (100.0) | 1.000 |
| Non-MF | 1 (1.7) | 0 (0.0) |  |
| **Major hepatectomy** |  |  |  |
| Yes | 14 (23.3) | 5 (23.8) | 0.965 |
| No | 46 (76.7) | 16 (76.2) |  |
| **Operative blood loss,** ml | 300.0 (100.0 – 400.0) | 150.0 (100.0 – 350.0) | 0.232 |
| **Blood transfusion** |  |  |  |
| Yes | 4 (6.7) | 1 (4.8) | 1.000 |
| No | 56 (93.3) | 20 (95.2) |  |
| **Surgical complication** |  |  |  |
| Yes | 16 (26.7) | 5 (23.8) | 0.797 |
| No | 44 (73.3) | 16 (76.2) |  |
| **Grade of complication** |  |  |  |
| I/II | 12 (64.2) | 3 (60.0) | 0.517 |
| III/IV | 4 (35.8) | 2 (40.0) |  |

**Abbreviations:** HBsAg, hepatitis B surface antigen; HBeAg, hepatitis B e antigen; HCV, hepatitis C virus; AFP, alpha-fetoprotein; CEA, carcinoembryonic antigen; CA19-9, carbonhydrateantigen19-9; TBIL, total bilirubin; ALB, albumin; ALT, alanine transaminase; AST, aspartate aminotransferase; ALP, alkaline phosphatase; PLT, platelet; PT, prothrombin time; MF, mass-forming; HR, hazard ratio; CI, confidence interval.
